# Consumption of Acidic Beverages is a Predisposing Factor for Erosive Tooth Wear in Preschool Children: A Population-based Study

**DOI:** 10.3290/j.ohpd.b871069

**Published:** 2020-12-14

**Authors:** Ananda Souza Pereira, Luciano Rodrigues Silva Lima, Marina de Deus Moura de Lima, Cacilda Castelo Branco Lima, Saul Martins Paiva, Lúcia de Fátima Almeida de Deus Moura, Marcoeli Silva de Moura

**Affiliations:** a Dentist, Department of Pathology and Dental Clinic, School of Dentistry, Federal University of Piauí, Teresina, Brazil. Experimental design, experiments on partial fulfillment of requirements for a Master’s degree, wrote the manuscript.; b Dentist, Department of Pathology and Dental Clinic, School of Dentistry, Federal University of Piauí, Teresina, Brazil. Experimental design.; c Professor, Department of Pathology and Dental Clinic, School of Dentistry, Federal University of Piauí, Teresina, Brazil. Proofread the manuscript, contributed substantially to discussion.; d Professor, Department of Pathology and Dental Clinic, School of Dentistry, Federal University of Piauí, Teresina, Brazil. Proofread the manuscript, performed tatistical analysis.; e Professor, Department of Paediatric Dentistry and Orthodontics, Federal University of Minas Gerais, Belo Horizonte, Brazil. Proofread the manuscript, contributed substantially to discussion.; f Professor, Department of Pathology and Dental Clinic, School of Dentistry, Federal University of Piauí, Teresina, Brazil. Proofread the manuscript, contributed substantially to discussion.; g Professor, Department of Pathology and Dental Clinic, School of Dentistry, Federal University of Piauí, Teresina, Brazil. Idea, hypothesis, experimental design, proofread the manuscript and contributed substantially to discussion.

**Keywords:** child, preschool, prevalence, tooth erosion

## Abstract

**Purpose:**

In a cross-sectional population-based study of 888 5-year-old preschoolers from Teresina, Brazil, to determine the prevalence and factors associated with erosive tooth wear (ETW).

**Materials and Methods:**

In a questionnaire, parents provided information on sociodemographic factors, their children’s eating/drinking habits, and oral health. Dental examination was performed at the schools by two calibrated examiners for the diagnosis of ETW, following the criteria of the modified O’Brien Index.

**Results:**

The prevalence of ETW was 3.3%. The maxillary incisors were the most highly affected teeth, followed by the mandibular and maxillary molars. The majority of the lesions reached only the enamel (72.1%) and up to 1/3 of the dental surface (63.1%). The occlusal surface was the most affected (47.8%). Preschoolers who had an acidic food-consumption profile had a 2.74 times (95% CI = 1.03–7.30) higher chance of having ETW than children without this profile.

**Conclusion:**

The prevalence of erosive tooth wear on the deciduous teeth was low and associated with the consumption of acidic beverages.

Changes in eating habits determined by the food and beverage industry, as well as gastrointestinal diseases, have sparked interest in erosive tooth wear (ETW).^18^ Chemomechanical wear results in the cumulative and irreversible loss of hard dental tissues.^6^ Acids of extrinsic (work environment, medications, acid drinks and fruits) or intrinsic sources (gastroesophageal reflux and vomiting) promote dental dissolution in the erosive process.^14^,^22^,^29^ Socioeconomic conditions are also mentioned as being associated with ETW.^19^,^21^,^29^

ETW may impair the development of masticatory and phonetic functions. It may be associated with dental sensitivity and aesthetic changes,^17^ which negatively affect the self-perception of affected individuals in terms of their oral health and quality of life.^22^

The reported prevalence of erosive tooth wear is variable in the deciduous dentition, with values ranging from 0.6% to 95%.^2^,^21^,^22^,^30^,^24^ Early diagnosis in this population is extremely important for the prevention and limitation of tissue damage, since deciduous teeth are more susceptible to dissolution/erosive wear than are permanent teeth.^18^,^24^

Erosive wear in the deciduous dentition can be a predisposing factor for erosion in the permanent dentition, since eating habits and health conditions during the deciduous dentition phase can be perpetuated throughout life.^11^

Given the above, this study aimed to determine the epidemiological profile and the factors associated with ETW in preschool children.

## Materials and Methods

### Ethical Aspects and Study Design

This cross-sectional study was approved by the Research Ethics Committee (CEP) of the Federal University of Piauí, Brazil (Approval: 2,085,469). The manuscript was written in accordance with the STROBE guidelines (Strengthening the Reporting of Observational Studies in Epidemiology).

### Study Design and Sample

This population-based study was developed with preschool children enrolled in public and private schools in Teresina, from September 2017 to May 2018. Teresina is the state capital of Piauí (northeast Brazil) and has an estimated population of 814,230 inhabitants, according to the Brazilian Institute of Geography and Statistics. In 2016, there were 10,213 five-year-old children enrolled in preschools in Teresina. Initially, a lottery was carried out to proportionally and randomly select the schools which would be participating in the study. Participation was requested through telephone contact.

The study included five-year-old preschoolers who had a complete deciduous dentition. This age was chosen because the children would be exposed to the predisposing factors investigated over a longer period. Children with special needs, palatal/lip clefts, amelogenesis imperfecta, a fixed orthodontic appliance, or who did not cooperate during the exam were excluded from the study.

### Sample Size Calculation

The sample size for this study was obtained using Epi-info software (Centers for Disease Control and Prevention; Atlanta, GA, USA), STATCALC module, v 7.0, using the formula: n = [EDFF * Np (1 – p)]/[(d2/Z21 – α/2 (N-1) + p * (1-p)]. The formula was adjusted by a correction factor of 1.5 (EDFF) for the study design, where N represents the population (10,213). A confidence interval of 95% (z21 – α/ 2 = 1.96) and a confidence limit (d) of 3% were considered. The proportion (p) of children with erosive tooth wear – 15.9% (n = 87) – obtained in a previous pilot study performed by this research group was considered, and a minimum sample of 812 preschool children was obtained. To compensate for possible losses, the sample size was increased by 20%, with a final sample of 974 (812 + 162 = 974) preschoolers. To ensure representativeness, the sample was stratified according to the region of the city (central-north, south, south-east and east) and the type of institution (public and private).

### Calibration Exercise

Calibration for the dental exam was conducted by a paediatric dentist with experience in epidemiological studies with a preschool population (standard of comparison). It was performed in three stages: 1) theory instruction for 4 h to present the index for the diagnosis of erosive wear, 2) the presentation of clinical cases with different degrees of severity, and 3) practical training on patients not participating in the study (n = 15). In these patients, a review was performed 15 days after the first evaluation. In this last step, it was possible to calculate the Kappa index of intra- and inter-examiner agreement for the two evaluators (intra-examiner Kappa for examiner 1 = 0.87; intra-examiner Kappa for examiner 2 = 0.81; inter-examiner Kappa 15.9% (n = 87) 1 –comparison pattern = 0.82, and inter-examiner Kappa 2 – comparison pattern = 0.86) of this study, obtaining a final value of 0.84 (inter-examiner Kappa 1– 2).

### Nonclinical Data Collection and Variables Studied

The socioeconomic data were collected through questionnaires sent and answered by parents/guardians of participating children, containing information on sex, family income (a Brazilian minimum wage equals 249.86 USD/month), maternal educational level (considering 8 years of schooling as formal basic education in Brazil) and oral hygiene habits. The health and dietary conditions cited as the factors possibly associated with dental erosion in previous studies were also investigated, including the presence of gastro-spastic reflux and vomiting, medication intake (vitamin C supplements, asthma inhalers containing corticosteroids and bronchial dilators, iron supplements), in addition to consumption frequency of acidic foods/beverages (soft drinks, fruits or acidic fruit juice) and protective foods/beverages (milk and yoghurt).

### Clinical Data Collection

The dental examination was carried out at the schools, after supervised oral hygiene with a toothbrush and fluoride dentifrice. The participant was in the simplified position (child’s head on the operator’s lap), and artificial lighting was used (Pelican model – Startec with 127V, São Paulo, Brazil). The examinations were performed using a plane oral mirror (Golgran; São Paulo, Brazil) and a CPI probe (Trinity, São Paulo Brazil).

The diagnostic criteria used for erosive tooth wear were those of the modified O’Brien index.^2^,^3^,^7^,^10^,^14^,^19^,^23^,^24^ All of the teeth were examined, and the lesions were classified according to the depth, area and location of the most serious lesion.

The depth was scored before the area, with scores of 0, 1, 2 or 3 as follows: 0 = without erosion, 1 = enamel injury, 2 = dentin lesion, and 3 = lesion close to the pulp (modification proposed by Murakami et al^22^). For the area, the lesions were given the follow scores: 0 = without erosion, 1 = injury involving up to 1/3 of the surface, 2 = injury involving up to 2/3 of the surface, and 3 = injury involving more than 2/3 of the surface. Teeth with a restoration or an extensive carious lesion covering the entire surface, as well as missing teeth, received a score of 9 (assessment could not be made) for depth. Evaluation of the area was not feasible even in the presence of small restorations or carious lesions, being also assigned a code 9.

The differential diagnosis was performed between erosive wear, abrasion and attrition lesions. The etiology and clinical features of these noncarious cervical lesions (NCCL) differe from those of erosion and were not included in the anamnesis and clinical examination data. Abrasion is caused by mechanical wear (e.g. traumatic toothbrushing) and affects the cervical region of the free surfaces of teeth. In contrast, attrition affects the occlusal and incisal surfaces due to dental contact during mastication; this contact may be physiological (normal masticatory activity) or pathological (bruxism). In general, teeth affected by erosive wear exhibited a loss of their natural contour and morphology. The lesions present with a smooth, shiny appearance and may or may not exhibit a translucent halo along the gingival margin. The posterior teeth initially exhibited small undercuts (cusp tips) that may evolve into a cup-like shape and, in extreme cases, pulp exposure.^6^,^20^

### Statistical Analysis

Data processing and statistical analysis were performed using SPSS for Windows, v 20.0 (Armonk, NY, USA). Descriptions of the absolute and relative frequencies of the variables were performed. The two-step cluster analysis was used to group the preschoolers according to the acid and protective food consumption profile. Preschoolers with a profile of acidic foods consumed soft drinks, fruit juice or acidic fruits. As for the protective foods, two groups were created; in one group, the predominant characteristic was consumption of milk and yoghurt once or twice a week, and the other group consisted of those who rarely drank milk and/or ate yoghurt.

The dependent variable, erosive tooth wear, was dichotomised as to its presence or absence, and its association with the independent variables was verified through a logistic regression model. Variables with p-values ≤ 0.20 in the bivariate analysis were included in the multiple logistic regression. Only the variables with a p-value < 0.05 remained in the final model. The odds ratio (OR) and 95% confidence interval were calculated. The level of statistical significance was set at p < 0.05.

## Results

A total of 888 preschoolers and their parents/guardians participated in this study (a response rate of 91.1%). A total of 126 preschoolers were excluded since 37 questionnaires were incomplete and 89 did not cooperate with the clinical examination and/or were not present on the day of evaluation.

The socioeconomic and demographic characteristics as well as clinical conditions of the participants are described in Table 1. The prevalence of dental erosion in the evaluated sample was 3.3% (Table 2).

**Table 1:** Socioeconomic, demographic and clinical characteristics of study participants (n = 888 preschoolers)

Variables	N	%
**Sex**		
Male	456	51.4
Female	432	48.6
**Type of preschool**		
Public	586	66.0
Private	302	34.0
**Mother’s years of schooling**		
< 8	113	12.7
8 – 11	547	61.6
> 11	228	25.7
**Family monthly income** *		
< 1 MW	190	22.5
1 – 3 MW	466	55.1
> 3 MW	189	22.4
**Erosive tooth wear**		
Present	29	3.3
Absent	859	96.7
**Caries experience**		
Present	376	42.3
Absent	512	57.7
Total	888	100.0

Family income (minimum wage [MW], 1 MW = $249.86).

*Missing information; participants refused to respond.

**Table 2:** Characterization of teeth with erosive tooth wear in relation to depth, extension and localization of the lesion (n = 111 teeth)

Variable	N	%
**Teeth with erosive wear**		
Present	111	0.6
Absent	17.649	99.4
**Affected teeth**		
Maxillary molars	25	22.5
Maxillary canines	20	18.1
Maxillary incisors	35	31.6
Mandibular canines	3	2.7
Mandibular molars	28	25.2
**Depth**		
Enamel	80	72.1
Dentin	27	24.3
Injury close to pulp	4	3.6
**Extension**		
Less than 1/3 of the surface	70	63.1
Between 1/3 and 2/3 of the surface	25	22.5
Greater than 2/3 of the surface	16	14.4
**Localization**		
Vestibular	2	1.8
Palatine/Lingual	32	28.8
Incisal	24	21.6
Occlusal	53	47.8

Table 2 shows the characteristics and distribution of erosive tooth wear. Of the total teeth examined (17,649), 111 (0.6%) teeth exhibited erosive wear. The maxillary incisors were the most frequently affected teeth, followed by molars. Most of the lesions occurred on the occlusal surface (47.8%), involving only the enamel (72.1%) and affecting up to 1/3 of the dental surface (63.1%).

In the final model, preschoolers with acidic food consumption had a 2.74x (OR = 2.74, 95%CI = 1.03 - 7.30) higher chance of having erosive tooth wear than preschool children who did not have this profile (Table 3).

**Table 3:** Association between erosive tooth wear and different variables (socioeconomic and demographic variables, caries experience, brushing/eating habits, medication intake and presence of reflux/vomiting), n = 888 preschoolers

Variables	Erosive tooth wear					
	Present	Absent	Adjusted OR	p-value	Adjusted OR	p-value
	n (%)	(n%)	(95% CI)		(95% CI)	
**Sex**						
Female	15 (3.5)	417 (96.5)	1.14 (0.54 – 2.38)	0.736		
Male	14 (3.1)	442 (96.9)	1			
**Mother’s years of schooling**						
< 8	5 (4.4)	108 (95.6)	2.06 (0.58 – 7.28)	0.260		
8 – 11	19 (3.5)	528 (96.5)	1.61 (0.59 – 4.36)	0.351		
> 11	5 (2.2)	223 (97.8)	1			
**Family monthly income** *						
< 1	5 (2.6)	185 (97.4)	0.82 (0.25 – 2.75)	0.753		
1 – 3	16 (3.4)	450 (96.6)	1.08 (0.42 – 2.81)	0.868		
> 3	6 (3.2)	183 (96.8)	1			
**Caries experience**						
No	21 (4.1)	491 (95.9)	1.97 (0.86 – 4.49)	0.108	2.09 (0.91 – 4.80)	0.083
Yes	8 (2.1)	368 (97.9)	1		1	
**Frequency of brushing (daily)**						
Once	6 (5.7)	100 (94.3)	1.98 (0.79 – 4.97)	0.147	–	–
Twice or more	23 (2.9)	758 (97.1)	1		–	
**Acidic food consumption profile**						
Present	24 (4.1)	562 (95.9)	2.54 (0.95 – 6.72)	0.061	2.74 (1.03 – 7.30)	0.044
Absent	5 (1.7)	297 (98.3)	1		1	
**Protective food consumption profile**						
Present	23 (4.0)	559 (96.0)	2.06 (0.83 – 5.11)	0.120	1.93 (0.77 – 4.82)	0.158
Absent	6 (2.0)	300 (98.0)	1		1	
**Medication intake related to erosion**						
Present	8 (4.4)	175 (95.6)	1.49 (0.65 – 3.42)	0.348		
Absent	21 (3.0)	684 (97.0)	1			
**Reflux/vomiting**						
Present	7 (5.4)	123 (94.6)	1.90 (0.80 – 4.55)	0.148		–
Absent	22 (2.9)	736 (97.1)	1		–	–

Familiy income (minimum wage - SM - 1SM = $ 249.86). * Data with missing information whose participants refused to respond. OR: Odds Ratio; CI: Confidence Interval; Hosmer & Lemeshow test = 0.764. Final model adjusted by carie experience and protective food consumption profile.

## Discussion

This population-based study investigated the prevalence of and factors associated with erosive tooth wear in preschoolers. The prevalence observed was low (3.3%) compared to the scientific literature (Table 4). Globally, a wide variation exists in the prevalence values of this pathology. Possible explanations for this are differences in the age groups studied, study designs, cultural, demographic and socioeconomic aspects, and especially the lack of a universally accepted standard diagnostic index.^17^,^31^

**Table 4:** Main studies published between 2002–2018 on prevalence of dental erosion in the deciduous dentition

Author (year)	Prevalence (%)	Country	Index	Sample	Age (years)	Associated factors
Al-Malik et al (2002)	31.3%	Saudi Arabia	O’Brien	987	2–5	Consumption of vitamin C, soda (1 or 2x/week) and fruit syrup when sleeping as a baby. Caries as a predictor of dental erosion.
Al-Majed et al (2002)	95%*/34%**	Saudi Arabia	O’Brien	354	5–6	Consumption of carbonated drinks (every night or from 4 to 6 nights/week).
Luo et al (2005)	5.7%	China	O’Brien	1949	3–5	High level of parental education. Intake of fruit juice by bottle or at bedtime.
Wiegand et al (2006)	32%	Germany	O’Sullivan	463	2–7	No related factors.
Taji et al (2010)	74/75/77%+	Australia	Modified TWI	128	2–4	No related factors.
Corrêa et al (2011)	25.2%	Brazil	O’Brien	232	2	Consumption of soft drinks, acidic sweets and gastric disorders. Milk consumption was considered a protective factor against erosion.
Murakami et al (2011)	51.6%	Brazil	Modified O’Brien	967	3–4	Consumption of acidic drinks (2 or 3x/day), gastroesophageal reflux and age.
Moimaz et al (2013)	0.6%	Brazil	TWI	1193	4–6	No related factors.
Fung and Messer (2013)	32%	Australia	Modified O’Brien	154 sub-sample	6	Consumption of fruit juice (two to four glasses/day).
Mantonanaki et al (2013)	78.8%	Greece	BEWE	524	5	Good oral hygiene. Low level of maternal schooling. High monthly family income.
Habib et al (2013)	13%	USA	TSL	164	2–4	Family income. Intake of acidic fruit juices (1x or more per week).
Tao et al (2015)	15.1%	China	O’Sullivan	1837	3–6	High maternal schooling. Occurrence of vomiting. Consumption of vinegar, coffee and tea; place of birth.
Al-Ashtal et al (2016)	93.2%*/6.8**	Yemen	EPRS and EPRS-M	206	5–6	No related factors.
Gopinath (2016)	58.5%	United Arab Emirates	O’Brien	403	5	Caries experience and consumption of acidic beverages.
Murakami et al (2016)	51.6% (2008), 53.9% (2010) and 51.3% (2012)	Brazil	Modified O’Brien	2801	3–4	No related factors.
Tschammler et al (2016)	31.3% (2004/05) to 45.4% (2014/15)	Germany	BEWE O’Sullivan	432 in 2004/05 compared to 775 in 2014/15	3–6	Age, sex and consumption of fruit juices or cocoa/lemonade.
Frazão et al (2018)	11.7%	Brazil	BEWE	239	6–10	Age and kind of school.
Duangthip et al (2019)	14.9%	China	BEWE	1204	3–5	Increase with age, low maternal educational level and poor oral hygiene.

*Erosion in enamel/ ** erosion in dentin/ + prevalence values recorded in dizygotic twins, non-twin controls and monozygotic twins, resp.

A systematic review reported the existence of nine tooth-wear indices applied in 29 studies to determine the prevalence of erosive wear in children and adolescents.^18^ However, the proposed diagnostic indices have some weaknesses. The TWI index (Tooth Wear Index) of Smith and Knight (1984) does not consider aetiology and does not distinguish between other forms of wear in scoring the assessed tooth.^4^ The BEWE index (Basic Erosive Wear Examination, 2007) only scores the teeth with the highest score in each sextant. This feature loses sight of the whole picture, but its main advantage is that this index assigns a risk rating to the patient.^4^ In addition, changes to the indices have been proposed, based on the clinical experience of some researchers, who sometimes apply the chosen index for groups of teeth and certain surfaces.^3^,^14^,^15^,^17^,^19^.

The present study used the O’Brien index,^25^ due to the possibility of evaluating all the teeth and dental surfaces, registering the one in the worst condition, and allowing the researchers to observe the effects on the entire dentition. The modification proposed by Murakami et al^23^ made the index more consistent with clinical reality than did the original index (O’Brien^25^), as pulp exposure is not commonly observed in eroded teeth. This index has proved to be adequate because of its reproducibility in epidemiological studies with children as well as its good comprehensibility and easy application.^10^,^18^,^23^,^24^

In this study, the teeth most affected by ETW were maxillary incisors, followed by molars. Maxillary incisors are more prone to erosive wear because they are more likely to be exposed to acids when patients eat and drink, or when reflux/vomiting occurs.^1^,^9^

Most of the lesions affected up to 1/3 of the dental surfaces; similar data were observed in studies evaluating the complete dentition using different indices.^21^,^31^ In this study, the lesions were limited to enamel; these results were also found in studies with a higher prevalence of erosive tooth wear.^1^,^2^,^10^,^21^,^31^

It is possible to observe that ETW was overestimated in some of the studies,^1^,^14^,^21^,^28^ a fact that is perhaps attributed to a lack of standardization of the diagnosis, making comparisons difficult.^27^ These difficulties were overcome in this study for a number of reasons: the index used is well established in the literature, all primary teeth were examined, and mainly because differential diagnosis between abrasion and attrition was performed. A consensus among specialists is necessary to overcome this limitation, to provide the best evidence to the community and to understand the impact of ETW on the deciduous dentition.

In this study, socioeconomic factors (family income and parental education level) were not found to influence ETW. However, there is no consensus of the association between erosive tooth wear, socioeconomic status and parents’ educational level.^8^,^15^,^19^,^21^

Although no association was found between erosive tooth wear, caries and toothbrushing frequency, in previous studies, these variables were associated with the highest level of wear observed,^14^,^21^ and caries was specifically considered a predictor for ETW.^3^

In this study, consuming vitamin C, using asthma inhalers containing corticosteroids and bronchial dilators, and taking iron supplements was not associated with erosive tooth wear. The data available on the influence of these substances on the development of erosive tooth wear reveal a weak level of evidence. It is suggested that the prolonged use of low-pH substances/medications may indirectly reduce the buffering capacity and flow of saliva, consequently increasing the risk of developing dental erosion.^5^ Another condition mentioned in previous studies^7^,^23^,^29^ to be associated with ETW was the presence of gastric reflux or vomiting. However, we found no association with the pathology in question,^21^ perhaps because the prevalence/frequency of these conditions was low in this study’s participants, so the exposure time was not sufficient to generate measurable clinical changes.

The acid consumption profile was the only variable associated with dental erosion in the present study. This variable increased the likelihood of ETW in children who consumed these foods/drinks. The acids present in these foods and drinks are generally considered weak. However, they possess a high buffering capacity at a specific pH, which maintains the dissolution process for longer periods.^5^ Only the studies by Wiegand et al^31^ and Frazão et al^9^ did not show an association between acidic beverages and ETW.

The contribution of a diet rich in acidic foods and drinks has already been highlighted in previous studies as a factor that increases the prevalence and severity of ETW.^3^,^14^,^19^,^30^ This factor can be modified or avoided if awareness of a healthy lifestyle is increased; then the progression of the lesions could be delayed and attributed to less preventable intrinsic acid sources.^26^ ETW is an important oral manifestation, because it can call attention to eating disorders,^16^ and signal the dental professional that intervention in a patient’s eating habits is necessary, considering that parents may not perceive this manifestation as a problem of oral health.^12^

The consumption of milk and yoghurt has been shown to have a protective effect against ETW.^7^ The main hypothesis supporting this idea is that the calcium and phosphate present in such foods can neutralize demineralization on the dental surface.^5^ This association was not observed in the current study, probably because the milk consumed by preschoolers was not directly consumed but was mixed with porridge or chocolate formulas, which may have interfered with the isolated evaluation of milk’s role in the erosive process.

The absence of factors associated with erosive tooth wear reinforces the fact that this pathology is multifactorial and that a wide range of complex factors and interactions should be considered when analyzing the development and progression of such lesions.^20^ In addition, memory bias may be another possible limitation of the results obtained, since the fidelity of data depended on parents’/guardians’ understanding of the questions asked, the ability to remember the required information and their desire to reveal the information.^13^

## Conclusion

ETW was not highly prevalent among the participants of this study, and was associated with the acid consumption profile. Parents should be vigilant about of their children’s eating habits and behaviors, because ETW can be an important indicator of/prelude to other problems. Considering the lower acid resistance and degree of mineralization of deciduous dentition, if deleterious habits are established, their maintenance in permanent dentition can compromise the health of these individuals.
